# Protective effect of fluoxetine against oxidative stress induced by renal ischemia-reperfusion injury via the regulation of miR-450b-5p/Nrf2 axis

**DOI:** 10.18632/aging.204289

**Published:** 2022-09-20

**Authors:** Zhiqiang Qin, Hao Wang, Quanliang Dou, Luwei Xu, Zheng Xu, Ruipeng Jia

**Affiliations:** 1 Department of Urology, Nanjing First Hospital, Nanjing Medical University, Nanjing 210006, China

**Keywords:** renal ischemia-reperfusion injury, fluoxetine, miR-450b-5p, Nrf2, oxidative stress

## Abstract

The present study was performed to assess the protective effect of fluoxetine (FLX) on renal ischemia-reperfusion injury (IRI) via the regulation of miR-450b-5p/Nrf2 axis in male rats. *In vivo*, these male rats were randomly divided into different treatment groups. The rats were administered with FLX (20 mg/kg, intraperitoneally) once daily for 3 days before operation. The pathomorphological changes of renal tissues were assessed by histological examination and Masson staining. *In vitro*, HK-2 cells were used to detect the activity by CCK-8 assay in Hypoxia/Reoxygenation (H/R) group and Hypoxia/Reoxygenation+Fluoxetine (H/R+FLX) group. In addition, the oxidative stress biomarkers were evaluated. Subsequently, Nrf2, NF-κB, and Nrf2-dependent antioxidant enzymes, were detected by Western blot assay. *In vivo*, the pathological changes and serological renal function were significantly relieved in the rats with the pre-treatment of FLX, compared to IRI group. After FLX stimulation, the expression levels of oxidative stress indices significantly decreased, while tissue antioxidant indices significantly increased, compared to IRI group. The differently expressed miRNAs on renal IRI in male rats were screened out by miRNA microarray, especially showing that miR-450b-5p was selected as the target miRNA. Following miR-450b-5p agomir injection, the pathological changes and oxidative stress biomarkers significantly aggravated, whether in IRI group or IRI+FLX group. Bioinformatics analysis and double-luciferase reporter assay demonstrated that miR-450b-5p directly targeted Nrf2. The expression level of NF-κB significantly increased, while the expression levels of Nrf2 and Nrf2-dependent antioxidant enzymes significantly decreased after miR-450b-5p agomir injection. Furthermore, the expression levels of Nrf2 and it-dependent antioxidant enzymes were apparently increased in ischemic kidney after the transfection of miR-450b-5p mimic+recombination protein Nrf2, as well as the decreased expression levels of intracellular ROS and iNOS. *In vitro*, FLX significantly increased HK-2 cell viability, and relieved H/R HK-2 cell oxidative injury via down-regulating ROS and iNOS. In addition, H/R-induced oxidative damage was recovered with miR-450b-5p mimic and recombination protein Nrf2. Consequently, FLX played an important protective role in renal IRI-induced oxidative damage by promoting antioxidation via targeting miR-450b-5p/Nrf2 axis.

## INTRODUCTION

Renal ischemia and reperfusion injury (IRI) is often caused by kidney surgery, trauma, shock or other injuries, which is a primary cause of acute organ dysfunction [1]. In a short period of time, renal IRI related acute kidney injury (AKI) is a common clinical problem that causes rapid decline in renal function [2]. The pathogenesis of renal IRI might be mediated by multiple mechanisms including oxidative stress, inflammatory responses, endothelial dysfunction, and so on [3, 4]. Although the exact etiology of renal IRI remains unclear and there is no effective treatment, the oxidative mediators released after the reperfusion injury are thought to be responsible for renal IRI [5]. When the blood supply is re-established, it will lead to excessive production of reactive oxygen species (ROS), thus resulting into the change of the properties of proteins, lipids, and ribonucleic acids in cells, which might cause cell dysfunction or even death [5, 6]. The necrotic cells might cause the activation of the oxidative stress and inflammatory cascade reaction, leading to more serious secondary tissue damage [7]. Therefore, it is of great significance to find an appropriate and reasonable preventive measure of AKI caused by renal IRI.

Recently, numerous investigations have shown that oxidative stress is one of the important factors in the occurrence and development of renal IRI [5–7]. Nuclear transcription factor erythroid 2-related factor 2 (Nrf2) is considered to be a key regulatory factor in the body's resistance to oxidative stress, which plays an important role in the protection of endothelial cell function [8]. In addition, the activation of Nrf2 can inhibit oxidative stress and inflammatory response, contributing to be an important way to treat renal ischemic disease by the repair of renal tubular epithelium [8, 9]. Fluoxetine (FLX), which belongs to a class of selective serotonin reuptake inhibitor (SSRI), is likely used to treat psychiatric conditions [10]. Recent studies have found that FLX meaningfully restored inflammation parameters and impaired redox balance subjected to IRI to baseline values [10, 11]. Thus, FLX might be thought of as a valid protective tactic via activation of Nrf2. However, it is still unknown whether by Nrf2 activation, FLX does administer to the protection of renal IRI.

microRNA (miRNA), as one of the small non-coding RNAs (ncRNAs), is involved in the maintenance of multiple organ functions, differentiation, proliferation, and immune system development by binding with target mRNA [12, 13]. In our study, according to the Gene Expression Omnibus (GEO) dataset, we have found that the expression level of miR-450b-5p significantly increased in ischemic kidney. An increasing number of investigations have also shown that miR-450b-5p plays an important role in a variety of diseases, suggesting that it might be considered as potential targets for the treatment of renal ischemic diseases [14, 15]. As far as we know, none of studies has as yet worked on oxidative stress modulating effects of FLX via the regulation of miR-450b-5p/Nrf2 axis on renal IRI both *in vitro* and *in vivo*.

In the present study, we tested the hypothesis that the pre-administration of FLX, as a preoperative anxiolytic, could prevent or attenuate renal oxidant and pro-inflammatory responses. Therefore, this study aimed to explore the protective effect of FLX on the progression of renal IRI by resisting oxidative stress responses and clarify the potential mechanism by which FLX modulated miR-450b-5p/Nrf2 axis.

## MATERIALS AND METHODS

### Animal experiments

The male 8-week-old Sprague-Dawley (SD) rats (200 ± 20 g) were purchased from the Animal Experiment Center of Nanjing Medical University (Nanjing, Jiangsu, China). Before the experiment, all animals were fasted for 24 hours and water was taken freely. The animal experiments were carried out in accordance with the experimental procedures approved by the Committee on the Ethics of Animal Research in Animal Care Facility of Nanjing First Hospital, Nanjing Medical University (approval number DWSY-1800505) (Nanjing, Jiangsu, China). Furthermore, the study was strictly in accordance with the recommendations in the Guide for the Care and Use of Laboratory Animals of the National Institutes of Health.

### Surgical procedure

Surgical procedures were performed by vascular clipping techniques, and were in narcotism by the intraperitoneal injection of pentobarbital (20 mg/kg, dissolved in 0.9% sodium chloride) (MedChemExpress, Monmouth Junction, NJ, USA). All SD rats with IRI were operated as follows: The dorsal median incision was firstly performed, and then the skin and subcutaneous layers were separated to the bilateral areas through blunt anatomy. Then, make a small incision on the right abdominal muscle, circumcise the right kidney, ligate the right renal artery, remove the right kidney, and then suture the muscular layer. The same operation was performed on the left side, but the left renal artery was clamped with a non-traumatic microvascular clamp (Roboz Surgical Instruments, Gaithersburg, MD, USA) for 45 minutes. These rats were randomly divided into six groups: (1) SHAM group, that only underwent the surgical procedure, without the clamping of the renal artery; (2) FLX group, received with FLX (20 mg/kg, intraperitoneally) (MedChemExpress, Monmouth Junction, NJ, USA) dissolved in 0.9% sodium chloride, once daily for 3 days, without the clamping of the renal artery; (3) IRI group, the left renal artery was clamped with a non-traumatic microvascular clamp for 45 minutes; (4) IRI+NC agomir group (NC agomir, forward, 5′-UUCUCCGAACGUGUCACGUTT-3′ and reverse, 5′-ACGUGACACGUUCGGAGAATT-3′, Ribobio, China), these rats were injected subcutaneously with 2 nmol NC agomir, after the establishment of renal IRI model; (5) IRI+miR-450b-5p agomir group (miR-450b-5p agomir, forward, 5′-UUUUGCAGUAUGUUCCUGAAUA-3′ and reverse 5′-UUCAGGAACAUACUGCAAAAUU-3′, Ribobio, China), these rats were injected subcutaneously with 2 nmol miR-450b-5p agomir, after the establishment of renal IRI model; and (6) IRI+miR-450b-5p agomir+Nrf2 group, these rats were injected subcutaneously with 2 nmol miR-450b-5p agomir and 10 nmol recombinant Nrf2, after the establishment of renal IRI model. Subsequently, close the surgical incision, and the analgesic buprenorphine (0.1 mg/kg) was injected subcutaneously. These rats were monitored until they recovered in a chamber on the heating pad. In addition, these animals maintained a normal weekly diet for 3 days after surgery.

### Histological examination

The renal tissue samples were fixed in 10% formalin for 24 h at room temperature. Then, the tissue samples were embedded in paraffin, and tissue blocks were sliced into 5 μm sections, to stain with hematoxylin and eosin (H&E, Sigma-Aldrich, St. Louis, MO, USA). Next, the changes in renal tissue structure were assessed by two blind investigators by a standard light microscope (Olympus BX-51, Tokyo, Japan). Renal tubule brush margin loss, cast formation, disruption and dilation of the tubule, and cell lysis are considered injuries. Light microscope was used to detect the injured renal tubules at five different points on the same histological sections.

### Masson staining

To estimate the area of fibrotic nephropathy, the renal tissue samples were also fixed with 10% formaldehyde, then decalcified, dehydrated, permeabilized with xylene, embedded in wax, and finally sliced into 5 μm thick sections. According to instructions provided by the manufacturer, the renal tissue sections were stained with Masson trichrome staining (Sigma-Aldrich, St. Louis, MO, USA), after rinsing with distilled water for 3 times. The sections were dehydrated with ethanol series, removed with xylene, fixed with neutral resin, and the images of stained sections were taken with a standard light microscope (Olympus BX-51, Tokyo, Japan). Red staining indicated normal and blue staining indicates renal fibrosis.

### Serum biochemical measurement

By the end of the experiment, the blood of these rats was collected from the inferior vena cava without any anticoagulant. The blood samples were centrifuged at 2000 g at 4°C for 10 min, followed by 8000 g at 4°C for 10 min. Then, serum was collected and frozen at −80°C until used. The commercially available clinical chemistry analyzer (Roche, Rotkreuz, Switzerland) was used to detect the serum creatinine (Scr) and blood urea nitrogen (BUN).

### Biochemical measurements

The blood samples were collected by using a vacuum collection tube and immediately frozen and transported to the laboratory. Then, the blood samples were centrifuged (−4°C, 3000 rpm, 10 min) to obtain plasma. In addition, the tissue was weighed and washed with 0.9% NaCl. Subsequently, plasma and tissue samples were stored at −80°C. According to corresponding detection kit (Jiancheng Bioengineering Institute, Nanjing, China), Malondialdehyde (MDA), total antioxidant capacity (T-AOC) and glutathione (GSH) were detected to test for the levels of oxidative stress. In addition, the remainder of the kidney was stored in formalin for histological evaluation.

### Detection of reactive oxygen species (ROS)

Briefly, the fresh kidney tissue from rats was immediately frozen and sliced into slices 5 μm thick sections, to stain with fluorescence *In Situ* Dihydroethidium (Sigma-Aldrich, USA). The kidney slices were placed in a dark and humid container with 1 μm DHE, and incubated at 37°C for 30 mins. The intracellular ROS production in renal tissues was assessed by performing the determination of intracellular superoxide levels under a standard fluorescence microscopy (Eclipse Ti-SR, Nikon Co., Japan), and measuring the density of the images in arbitrary units per millimeter square by using a standard fluorescence spectrophotometer.

### Cell culture and transfection

The human renal proximal tubule epithelial cell line (HK-2), purchased from the Shanghai Institutes for Biological Sciences (Shanghai, China), was cultured in DMEM medium (HyClone, USA) supplemented with 10% fetal bovine serum (FBS, Gibco, CA, USA), 100 U/mL penicillin, and 100 μg/mL streptomycin at 37°C with 5% CO_2_ in a humidified atmosphere. The expression level of miR-450b-5p was up-regulated or down-regulated by transfection. miR-450b-5p mimic and NC mimic or miR-450b-5p inhibitor and NC inhibitor were dissolved in Opti-MEM, respectively, and these mixed solutions were equilibrated at room temperature for 5 mins. Subsequently, each solution was then mixed with the Lipofectamine 3000 transfection reagent, and gently mixed to form the inhibitor liposome for 20 mins. The transfected HK-2 cells were cultured in serum-free cell culture medium containing the transfection mixture at 37°C, 5% CO_2_ and 95% O_2_, and the mixed medium was replaced with fresh medium after 6 h.

### Cell hypoxic/reoxygenation (H/R) model

According to the manufacturer’s protocol, HK-2 cells were placed into a six-well plate in a hypoxic chamber with 94% N_2_, 5% CO_2_, and 1% O_2_ at 37°C for 2 h. Then, the HK-2 cells were cultured with hypoxia treatment at 0.5% O_2_ for 15 h, and were further cultured with constant oxygen for 6 h. In addition, HK-2 cells were incubated under normoxic conditions served as controls.

### Immunofluorescence assay

The renal tissues and HK-2 cells were fixed with 4% paraformaldehyde at room temperature for 10 mins and washed five times with PBS. The main antibodies were incubated at 4°C for a night and then incubated with Alexa fluorescin-labeled secondary antibody for 2 h at room temperature. DAPI staining was performed for 5 mins, and then the nuclei were observed with a fluorescence microscope (Eclipse Ti-SR, Nikon, Japan) at 400× magnification.

### Cell viability assay

The transfected HK-2 cells were seeded into 96-well plates at the density of 3000 cells per well. Cells were fostered in 5% CO_2_ incubator at at 37°C, 5% CO_2_ and 95% O_2_. After 0, 24, 48, 72 and 96 hours, 20 μL of CCK-8 solution (Dojindo Laboratory, Kumamoto, Japan) was added into each well, and then cells were continuously fostered for 4 h. The absorbance was measured at a wavelength of 450 nm and the cell growth curve was drawn.

### Fluorescence *in situ* hybridization (FISH)

Firstly, the renal tissues and HK-2 cells were fixed in 4% formaldehyde for 15 mins. Then, the fixed tissues and cells were washed with PBS, treated with pepsin, dehydrated with ethanol, and further incubated in hybridization buffer with 40 nm FISH probe. After hybridization, the sections were washed and dehydrated, and the nucleic acid was detected with DAPI and Prolong Gold Antifade Reagent. The slides were observed by immunofluorescence with Olympus confocal laser scanning microscope.

### Reverse transcription and quantitative PCR

The renal tissues and HK-2 cells were used to extract total RNA by using TRIzol kit. TRIzol was used to lyse each sample, and total RNA was extracted. PrimeScript RT kit (Takara, Dalian, China) was manufactured for reverse-transcription from total RNA to cDNA, and SYBR Green PCR Master Mix (Takara, Dalian, China) was used for quantitative PCR. In addition, the All-in-One™ miRNA qRT-PCR Detection Kit (Vazyme Biotech, Nanjing, China) was used to detect the expression level of miRNA by an ABI 7500 system (Applied Biosystems, Foster City, CA, USA). The fold changes at these genes were measured using 2−ΔΔCT method with U6 or GAPDH as the standard. The following primers were used for qRT-PCR:

miR-450b-5p, forward 5′-CGCGTTTTGCAGTATGTTCC-3′; reverse 5′-AGTGCAGGGTCCGAGGTATT-3′; reverse transcription PCR: CTCAACTGGTGTCGTGGAGTCGGCAATTCAGTTGAGTATTCAGG U6, forward 5′-CTCGCTTCGGCAGCACA-3′; reverse 5′-AACGCTTCACGAATTTGCGT-3′; Universal reverse TGGTGTCGTGGAGTCG Nrf2, forward 5′-AGGTTGCCCACATTCCCAAA-3′; reverse 5′-ACGTAGCCGAAGAAACCTCA-3′ FOXN3, forward 5′-AGATACAAGCAGGTTTTCCTCCA-3′; reverse 5′-CTCACTCTCAGTCCGCATCC-3′ PLK4, forward 5′-TTCCGTGGTTTCAGCGTC-3′; reverse 5′-TTTCCAACTTTAAAATCCTCGATCT-3′ FAN1, forward 5′-GATCTTGGGTGACAGGGCA-3′; reverse 5′-GACAATTTCTTCTTCCTGGGGC-3′ GAPDH, forward 5′-CTCTGCTCCTCCTGTTCGAC-3′; reverse 5′-GCGCCCAATACGACCAAATC-3′.

### Western blot

The renal tissues or transfected HK-2 cells were lysed with cell lysis buffer, shaken with ice for 30 mins, centrifuged at 4°C for 14000 × g for 15 mins. Total protein extraction kit was used to extract total protein (Active Motif, Tokyo, Japan). In this study, the primary antibodies against Nrf2 (97 kDa), NF-κB (65 kDa), heme oxygenase-1 (HO-1, 28 kDa), NADPH quinine oxidoreductase-1 (NQO-1, 29 kDa), and Glutathione Stransferase (GST, 51 kDa) were used (Cell Signaling Technology, Danvers, MA, USA). We used Histone H3 and GAPDH (Cell Signaling Technology, USA) as protein controls to normalize the expression levels of these proteins. Image Lab software (Bio-Rad, Hercules, CA, USA) was used for quantitative density analysis of protein bands.

### RNA immunoprecipitation (RIP)

According to the manufacturer’s instructions, RIP assay was performed by using Magna RIP RNA-Binding Protein Immunoprecipitation Kit (Millipore, Burlington, MA, USA). Finally, the magnetic bead-protein complexes were collected, digested by protease K, and RNA was purified and detected by qRT-PCR.

### Statistical analysis

In this study, Prism 7.0 software (GraphPad, San Diego, CA, USA) was used to analyze the data. One-way analysis of variance (ANOVA) was used to compare the differences between different groups. The experimental results from at least three independent trials (mean ± standard deviation); and *P* < 0.05 was considered statistically significant.

## RESULTS

### Fluoxetine prevented renal IRI in male rats

**Figure 1:**
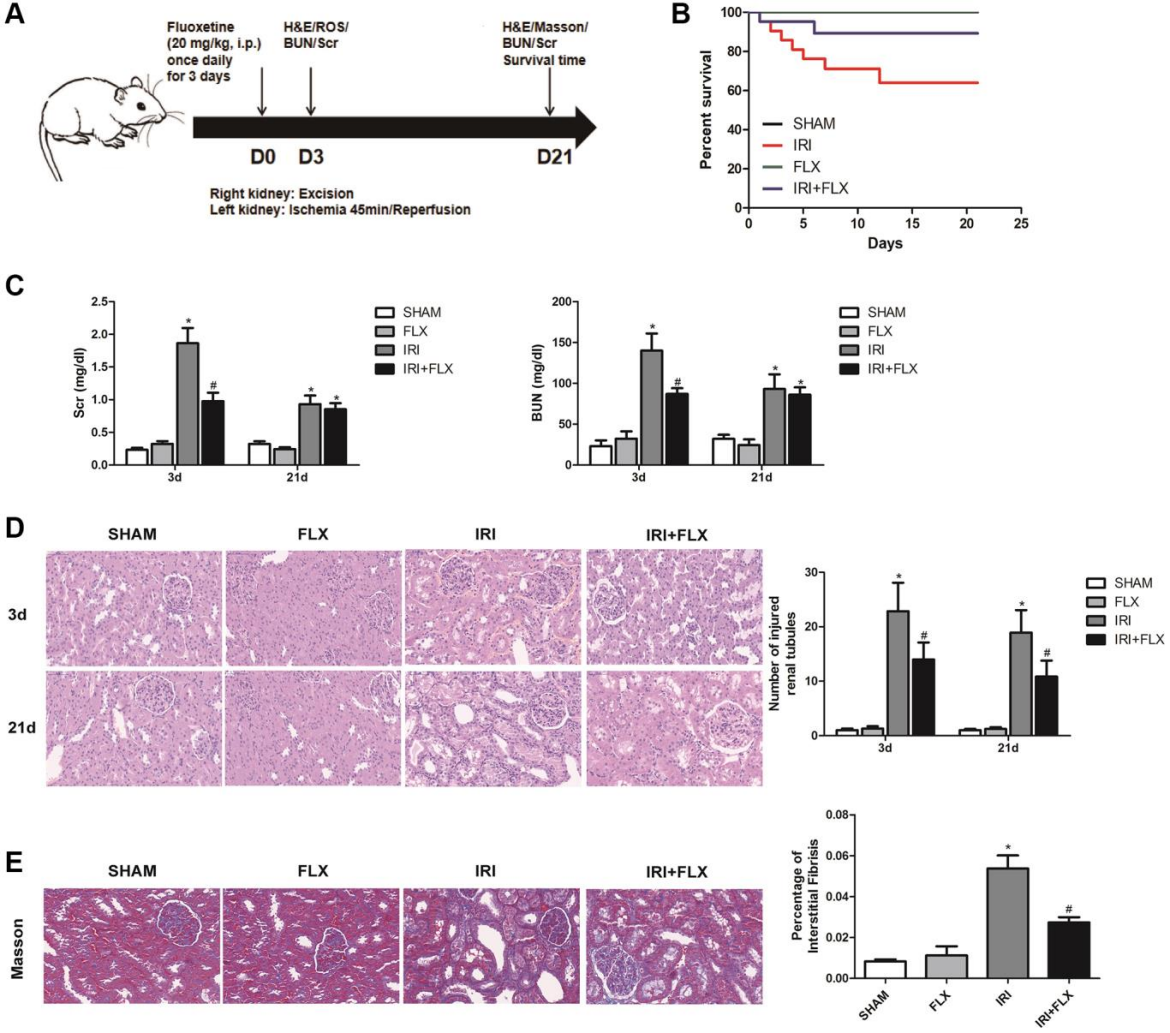
**Fluoxetine prevented renal IRI injury in male rats.** Fluoxetine prevented renal IRI in male rats. (**A**) Diagram of the renal IRI animal model. (**B**) Survival curves for SHAM, FLX, IRI and IRI+FLX groups. (**C**) Serum creatinine (Scr) and blood urea nitrogen (BUN) of blood sample collected at 24 h reperfusion from SHAM group, FLX group, IRI group, and IRI+FLX group. (**D**) H&E staining of renal tissues in SHAM, FLX, IRI, and IRI treated with Fluoxetine rats at 3 and 21 days after operation (magnification ×400). (**E**) Masson staining was used to evaluate renal injury and fibrosis. Mean seminiferous tubular diameter (magnification ×400). Data were presented as Mean ± SD, ^*^significant difference vs. SHAM group (*P* < 0.05); ^#^significant difference vs. IRI group (*P* < 0.05).

As shown in Figure 1A, the renal IRI rat model was established, and the relevant detection indexes were collected at corresponding time. Compared to SHAM group, FLX group and IRI+FLX group, the survival rate of these rats significantly decreased (*P* < 0.05, Figure 1B); and the levels of BUN and Scr at 3 days after operation significantly increased in IRI intervention group (*P* < 0.05, Figure 1C). In addition, renal IRI resulted into the renal tubule cell atrophy and dilatation of rats (*P* < 0.05; Figure 1D). Next, we prolonged the experiment time, and further observed the improved tubular injury as well as interstitial fibrosis on 21 days after renal IRI by Masson staining. We found that the ischemia of the kidney could cause *in situ* loss of parenchymal cells (*P* < 0.05; Figure 1E). The above results showed that FLX administration could protect these rats from renal IRI and subsequent early fibrotic injury.

### Fluoxetine alleviated oxidative damage caused by renal IRI in male rats

**Figure 2:**
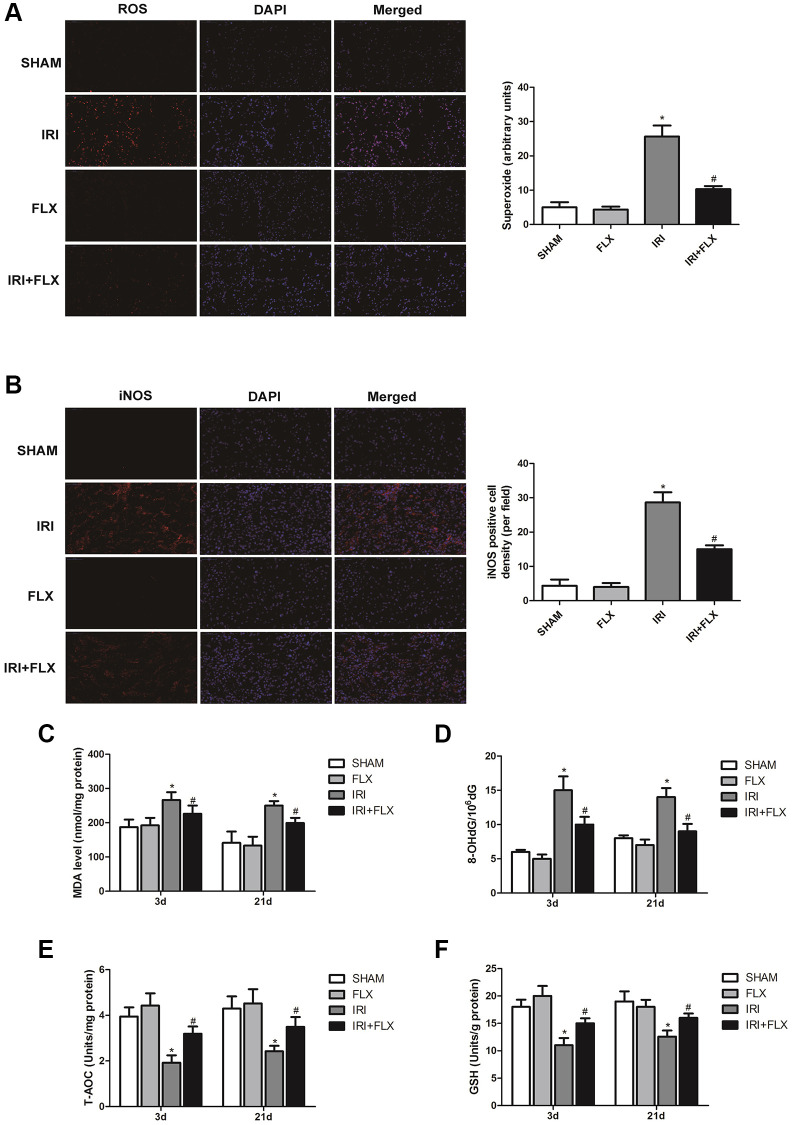
**Fluoxetine alleviated oxidative damage caused by renal IRI in male rats.** (**A**) DHE staining of renal tissues in SHAM, FLX, IRI and IRI+FLX groups. ROS exhibit red fluorescence under fluorescent microscope (magnification ×400). (**B**) Immunofluorescence Assay showed the expression of iNOS from renal ischemia tissues in each group (magnification ×400). (**C**–**F**) Content of MDA, 8-OHdG, T-AOC, and GSH of renal ischemia tissues in each group. Data were presented as Mean ± SD, ^*^significant difference vs. SHAM group (*P* < 0.05); ^#^significant difference vs. IRI group (*P* < 0.05).

Compared with SHAM group, the intensity of the fluorescent signals about ROS in the kidney tissues significantly increased in IRI group. In contrast, FLX could significantly reduce the intensity of the fluorescent signals of ROS, indicating that FLX could reduce oxidative stress induced by IRI in these rats (Figure 2A). As shown in Figure 2B, immunofluorescence assay also showed that the number of iNOS cells in IRI group was significantly higher than that in SHAM group; Nevertheless, the percentage of iNOS cells in FLX pretreatment group was significantly lower than that of IRI group (*P* < 0.05), which provided the evidences that FLX could protect against renal IRI-induced oxidative damage. As shown in Figure 2C–2F, the content of MDA and 8-OHdG in IRI group were significantly higher than these in SHAM group (*P* < 0.05); However, the pretreatment of FLX could significantly rescue the parameters, compared to IRI group (*P* < 0.05). In addition, FLX could increase the levels of antioxidants, including T-AOC and GSH, thus reducing renal IRI-induced oxidative stress; Nevertheless, these parameters in IRI group were significantly lower than these in SHAM group, thus indicating that FLX could alleviate renal IRI-induced oxidative damage *in vivo*.

### miR-450b-5p was high-expressed on renal IRI in rats

**Figure 3:**
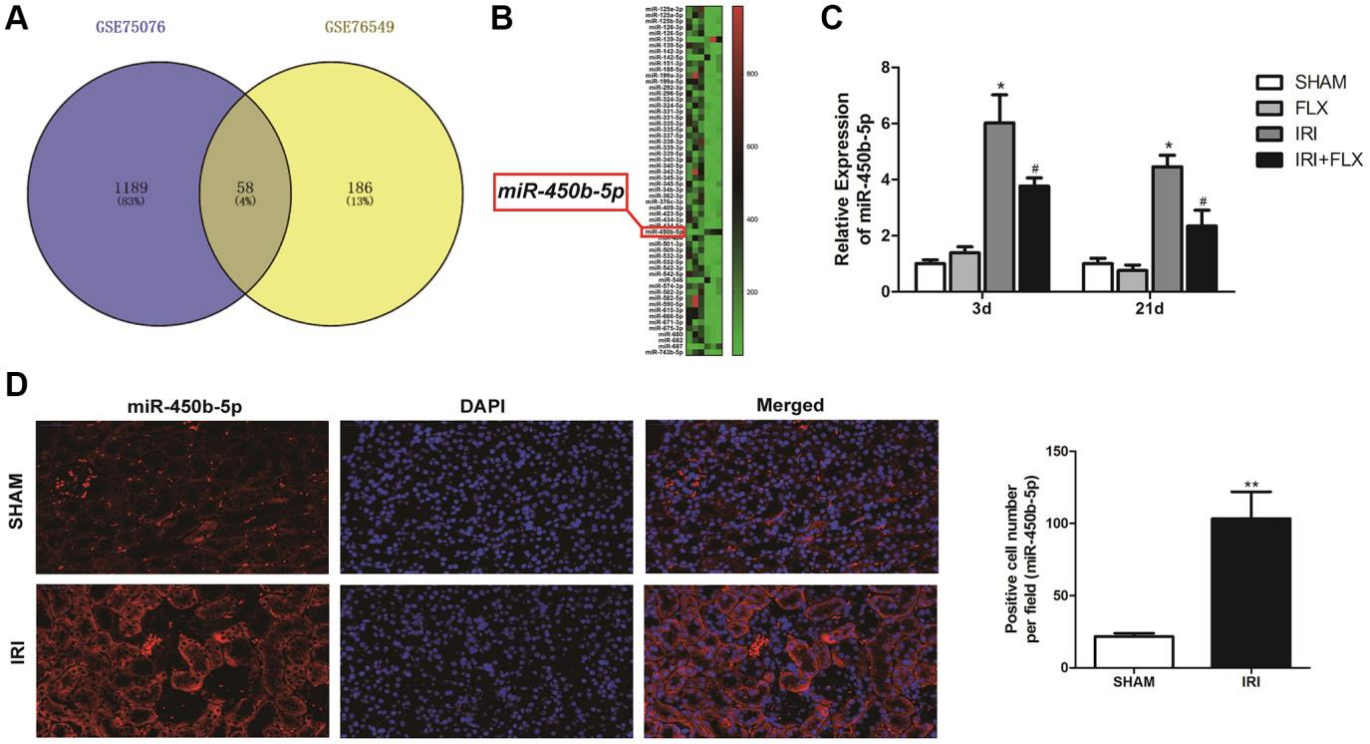
**miR-450b-5p was high-expressed on renal IRI in rats.** (**A**) The Gene Expression Omnibus (GEO) dataset obtained the potential differences of miRNAs by the gene expression matrices GSE75076, and GSE76549. (**B**) The heat map indicated the results of a two-way hierarchical clustering of the samples and found 58 differentially expressed miRNAs. (**C**) qRT-PCR assay showed the mRNA expression level of miR-450b-5p in SHAM, FLX, IRI and IRI+FLX groups. (**D**) FISH results showed the expression level of miR-450b-5p in SHAM group, and IRI group (magnification ×400). Data were presented as Mean ± SD, ^*^significant difference vs. SHAM group (*P* < 0.05; ^**^P < 0.01); ^#^significant difference vs. IRI group (*P* < 0.05).

After a series of data pre-processing steps from the gene expression matrices GSE75076 and GSE76549 based on the Gene Expression Omnibus (GEO) dataset, the results combined the gene expression matrices were analysed to find a total of the differentially expressed miRNAs in renal tissues caused by IRI of rats (Figure 3A). As shown in Figure 3B, the heat map indicated that the expression difference of miR-450b-5p was the largest, which was selected as the target miRNA. The expression level of miR-450b-5p significantly increased in IRI group, compared to SHAM group. Meanwhile, FLX could significantly decrease the expression level of miR-450b-5p in renal IRI-induced oxidative damage (*P* < 0.05) (Figure 3C). According to FISH results shown in Figure 3D, compared to that of SHAM group, the expression level of miR-450b-5p also significantly increased in renal tissues of IRI group.

### Up-regulation of miR-450b-5p aggravated oxidative stress in renal IRI-treated rats

**Figure 4:**
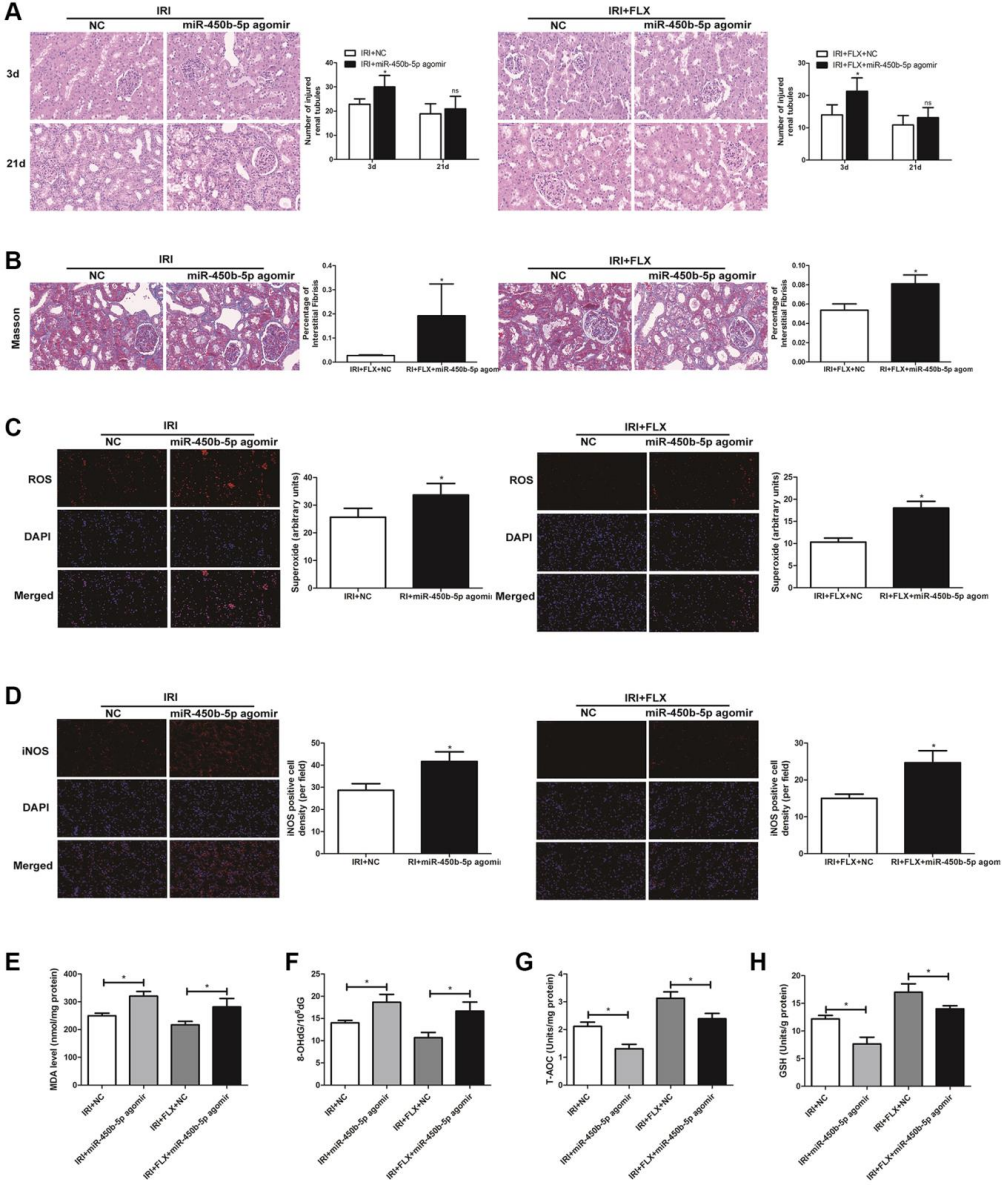
**Up-regulation of miR-450b-5p aggravated oxidative stress in renal IRI-treated rats.** (**A**) H&E staining of renal tissues in IRI group and IRI+FLX group after the injection of NC agomir and miR-450b-5p agomir at 3 and 21 days after operation in male rats (magnification ×400). (**B**) Masson staining was used to evaluate renal injury and fibrosis in IRI group and IRI+FLX group after the injection of NC agomir and miR-450b-5p agomir at 21 days after operation in male rats (magnification ×400). (**C**) DHE staining of renal tissues in IRI group and IRI+FLX group after the injection of NC agomir and miR-450b-5p agomir. ROS exhibit red fluorescence under fluorescent microscope (magnification ×400). (**D**) Immunohistochemical analysis showed the expression level of iNOS from renal tissues in IRI group and IRI+FLX group after the injection of NC agomir and miR-450b-5p agomir in male rats (magnification ×400). (**E**–**H**) Content of MDA, 8-OHdG, T-AOC, and GSH of renal ischemia tissues in each group. Data were presented as Mean ± SD, ^*^significant difference (*P* < 0.05; ^**^P *<* 0.01).

Following miR-450b-5p agomir injection, H&E staining showed that up-regulation of miR-450b-5p significantly aggravated the IRI-induced histological alterations, such as the obvious renal tubule dilatation and cell atrophy; and increased the number of tubule/unit area in IRI-operated rats. In addition, the intervention of FLX also reduced the intensity of inflammation, thus protecting renal IRI by histopathology to some extent (Figure 4A). Masson staining also showed that the supplement of FLX could reverse the loss of parenchymal cells and fibrotic collagen deposition, thus resulting into a weight loss of the injured kidney (Figure 4B). ROS staining was used to examine the intensity of the fluorescent signals about ROS in the kidney. Compared with IRI group, the high fluorescent signals of ROS were detected in IRI+FLX group, and massively accumulated in the renal medullary interstitium. Meanwhile, the intensity of the fluorescent signals about ROS significantly increased in the transfection of miR-450b-5p agomir, compared with NC agomir. Thus, these results suggested that the pretreatment of FLX might resist oxidative stress by decreasing the recruitment of miR-450b-5p in renal IRI rats (Figure 4C). Immunohistochemical analysis of iNOS also reflected this trend (Figure 4D). As shown in Figure 4E–4H, whether in IRI group or IRI+FLX group, miR-450b-5p agomir could obviously increase the expression levels of tissue MDA and 8-OHdG, while tissue antioxidant indices including the expression levels of T-AOC and SOD were significantly decreased, compared to NC agomir.

### Nrf2 is a direct target of miR-450b-5p

**Figure 5:**
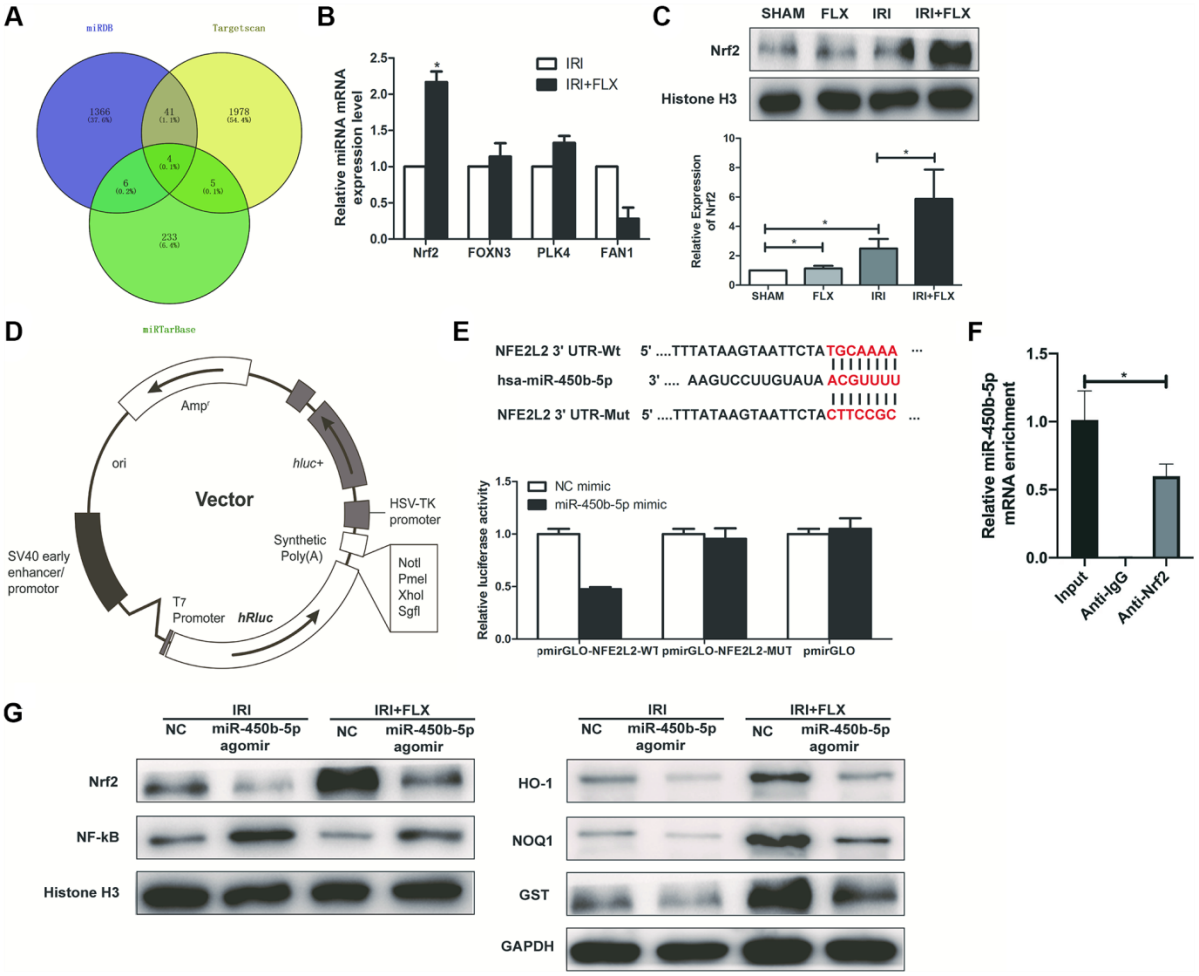
**Nrf2 is a direct target of miR-450b-5p on renal IRI in male rats.** (**A**) A schematic diagram used to search the target mRNAs of miR-450b-5p in three databases (MiRDB, StarBase and TargetScan). (**B**) Validation of the four differently expressed mRNAs in rats caused by IRI injury based on qRT-PCR assay. (**C**) Western Blotting analysis showed the expression level of Nrf2 from renal tissues in SHAM, FLX, IRI and IRI+FLX groups. (**D**) The construction diagram of the target genes (Nrf2) double-luciferase reporter genes. (**E**) The relative luciferase expression with Nrf2 3′-UTR after co-transfection with miR-450b-5p mimic or NC mimic in HK-2. (**F**) Nrf2 could bind to miR-450b-5p transcript *in vivo* in HK-2 cells. HK-2 cell lysates were immunoprecipitated with Nrf2 antibody or control IgG followed by qRT-PCR. (**G**) Western Blotting analysis showed the expression level of NF-κB, Nrf2 and Nrf2-dependent antioxidant enzymes, including HO-1, NQO1 and GST from renal tissues in IRI group and IRI+FLX group after the injection of NC agomir and miR-450b-5p agomir. Data were presented as Mean ± SD, ^*^significant difference (*P* < 0.05).

As shown in Figure 5A, Targetscan (http://www.targetscan.org/), MiRDB (http://www.mirdb.org/) and MiRTarBase were used to predict the interaction networks between the miR-450b-5p and its downstream mRNAs. Then, 4 potential mRNAs, including Nrf2, FOXN3, PLK4 and FAN1, were further validated by qRT-PCR assay. As shown in Figure 5B, the expression level of Nrf2 significantly increased in the ischemic kidney tissues of these rats by the administration of FLX. Western Blot assay showed that compared with SHAM group or FLX group, IRI group could slightly increase the protein expression level of Nrf2; Besides, IRI+FLX group significantly increased the protein expression level of Nrf2, to contribute to the resistance to oxidative stress caused by renal IRI (Figure 5C). We found that the 3′-UTR of Nrf2 mRNA contained a complementary site for the seed region of miR-450b-5p by the RNA sequence alignment (Figure 5D). As presented in Figure 5E, the dual-luciferase reporter assay showed that the luciferase activities were significantly repressed by miR-450b-5p mimic compared with NC mimic group in the groups of Nrf2-Wt. However, these effects were not observed in the groups of mutated Nrf2, suggesting that Nrf2 was the target gene of miR-450b-5p. Next, RIP assay was performed to validate whether Nrf2 could bind to miR-450b-5p transcript. HK-2 cells were immunoprecipitated with Nrf2 antibody or control rabbit IgG. RIP analysis demonstrated that miR-450b-5p transcript was significantly enriched in the Nrf2 immunocomplexes, but not in the control IgG immunocomplexes (Figure 5F). Subsequently, the expression level of NF-κB was significantly higher, while the expression levels of Nrf2 and Nrf2-dependent antioxidant enzymes, including HO-1, NQO1 and GST, were significantly lower after miR-450b-5p agomir injection, whether in IRI group or IRI+FLX group (Figure 5G).

### Fluoxetine pre-treatment relieved oxidative stress via miR-450b-5p/Nrf2 axis in renal IRI rats

**Figure 6:**
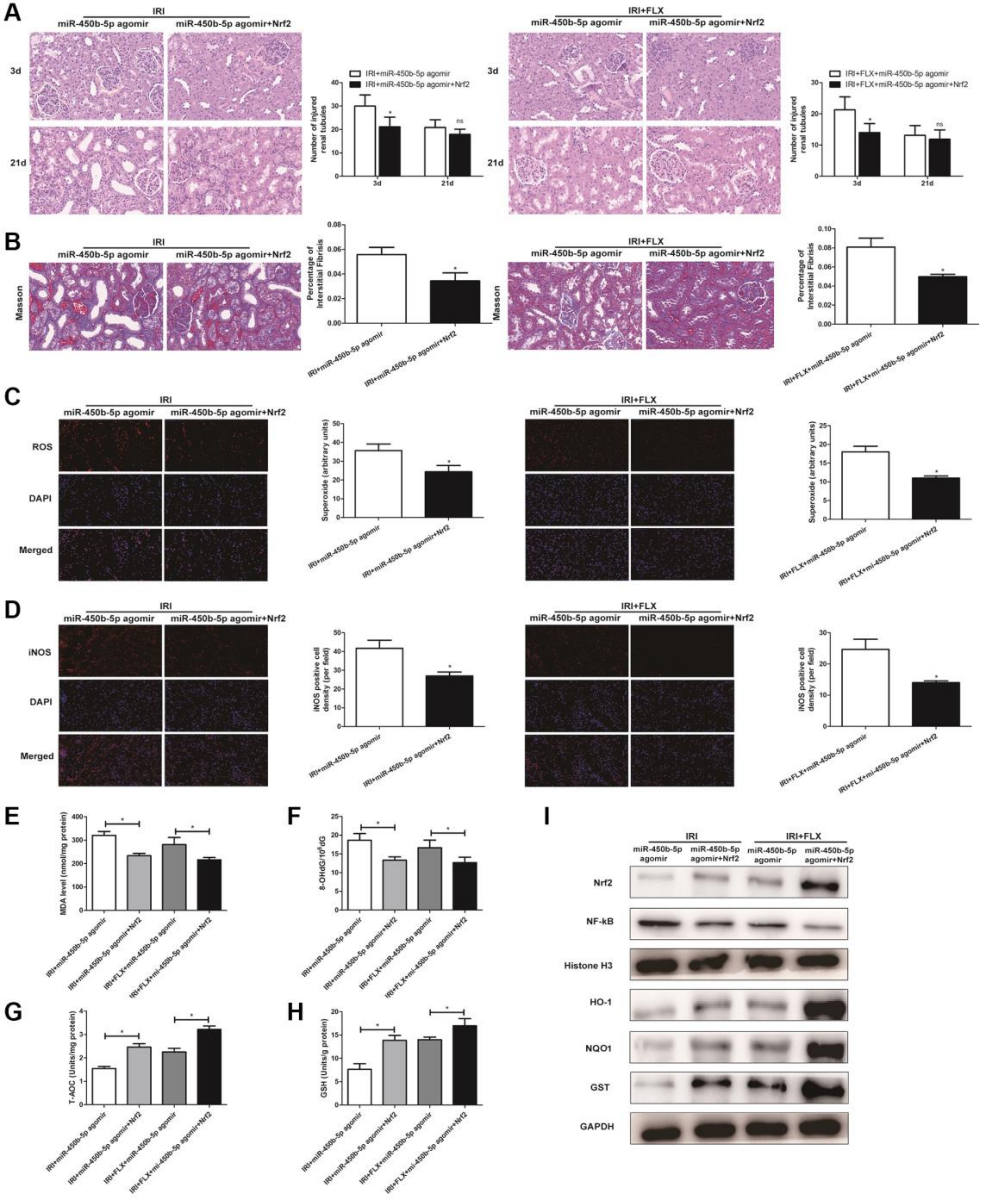
**Fluoxetine pre-treatment relieved oxidative stress via miR-450b-5p/Nrf2 axis in renal IRI rats.** (**A**) H&E staining of renal tissues in IRI group and IRI+FLX group at 3 and 21 days after operation after the injection of miR-450b-5p agomir or miR-450b-5p agomir+Recombinant protein Nrf2 in male rats (magnification ×400). (**B**) Masson staining was used to evaluate renal injury and fibrosis in IRI group and IRI+FLX group after the injection of miR-450b-5p agomir or miR-450b-5p agomir+Recombinant protein Nrf2. (**C**) DHE staining of renal tissues in IRI group and IRI+FLX group after the injection of miR-450b-5p agomir or miR-450b-5p agomir+Recombinant protein Nrf2. ROS exhibit red fluorescence under fluorescent microscope (magnification ×400). (**D**) Immunohistochemical analysis showed the expression level of iNOS from renal tissues in IRI group and IRI+FLX group after the injection of miR-450b-5p agomir or miR-450b-5p agomir+Recombinant protein Nrf2 in male rats (magnification ×400). (**E**) Content of MDA of renal ischemia tissues in IRI group and IRI+FLX group after the injection of miR-450b-5p agomir or miR-450b-5p agomir+Recombinant protein Nrf2. (**F**) Content of 8-OHdG of renal ischemia tissues in IRI group and IRI+FLX group after the injection of miR-450b-5p agomir or miR-450b-5p agomir+Recombinant protein Nrf2. (**G**) Content of T-AOC of renal ischemiatissues in IRI group and IRI+FLX group after the injection of miR-450b-5p agomir or miR-450b-5p agomir+Recombinant protein Nrf2. (**H**) Content of GSH of renal ischemia tissues in IRI group and IRI+FLX group after the injection of miR-450b-5p agomir or miR-450b-5p agomir+Recombinant protein Nrf2. (**I**) Western Blotting analysis showed the expression level of NF-κB, Nrf2 and Nrf2-dependent antioxidant enzymes, including HO-1, NQO1 and GST from renal tissues in IRI group and IRI+FLX group after the injection of miR-450b-5p agomir or miR-450b-5p agomir+Recombinant protein Nrf2. Data were presented as Mean ± SD, ^*^significant difference (*P* < 0.05).

H&E staining showed that miR-450b-5p agomir+Recombinant Nrf2 injection significantly relieved the histological alterations in renal IRI rats with miR-450b-5p agomir injection (Figure 6A). Moreover, Masson staining showed that the fibrotic collagen deposition were significantly decreased in miR-450b-5p agomir+Recombinant Nrf2 group, relative to miR-450b-5p agomir group; Besides, the intervention of FLX also significantly decreased the fibrotic collagen deposition, compared with IRI rats (Figure 6B, *P* < 0.05). In addition, ROS staining showed that miR-450b-5p agomir+Recombinant Nrf2 could decrease the intensity of the fluorescent signals, compared to miR-450b-5p agomir group, whether in IRI group or IRI+FLX group (Figure 6C). Immunohistochemical analysis also showed that, miR-450b-5p agomir+Recombinant Nrf2 could decreased the expression level of iNOS, compared to miR-450b-5p agomir group (Figure 6D). In addition, We found that the content of MDA (Figure 6E) and 8- OHdG (Figure 6F) significantly decreased, but the content of antioxidants, including T-AOC (Figure 6G) and GSH (Figure 6H), just significantly increased in miR-450b-5p agomir+Recombinant Nrf2 group, compared with miR-450b-5p agomir group following reperfusion, whether in IRI group or IRI+FLX group. As shown in Western Blot assay, the expression levels of Nrf2 and Nrf2-dependent antioxidant enzymes, including HO-1, NQO1 and GST, significantly increased in miR-450b-5p agomir+Recombinant Nrf2 group, compared to miR-450b-5p agomir group following reperfusion, whether in IRI group or IRI+FLX group (Figure 6F). Thus, these results suggested that the pretreatment of FLX might promote antioxidant ability by decreasing the recruitment of miR-450b-5p/Nrf2 axis in renal IRI-treated rats.

### Fluoxetine up-regulated miR-450b-5p via Nrf2 pathway in HK-2 cells Hypoxia/Reoxygenation (H/R) model

**Figure 7:**
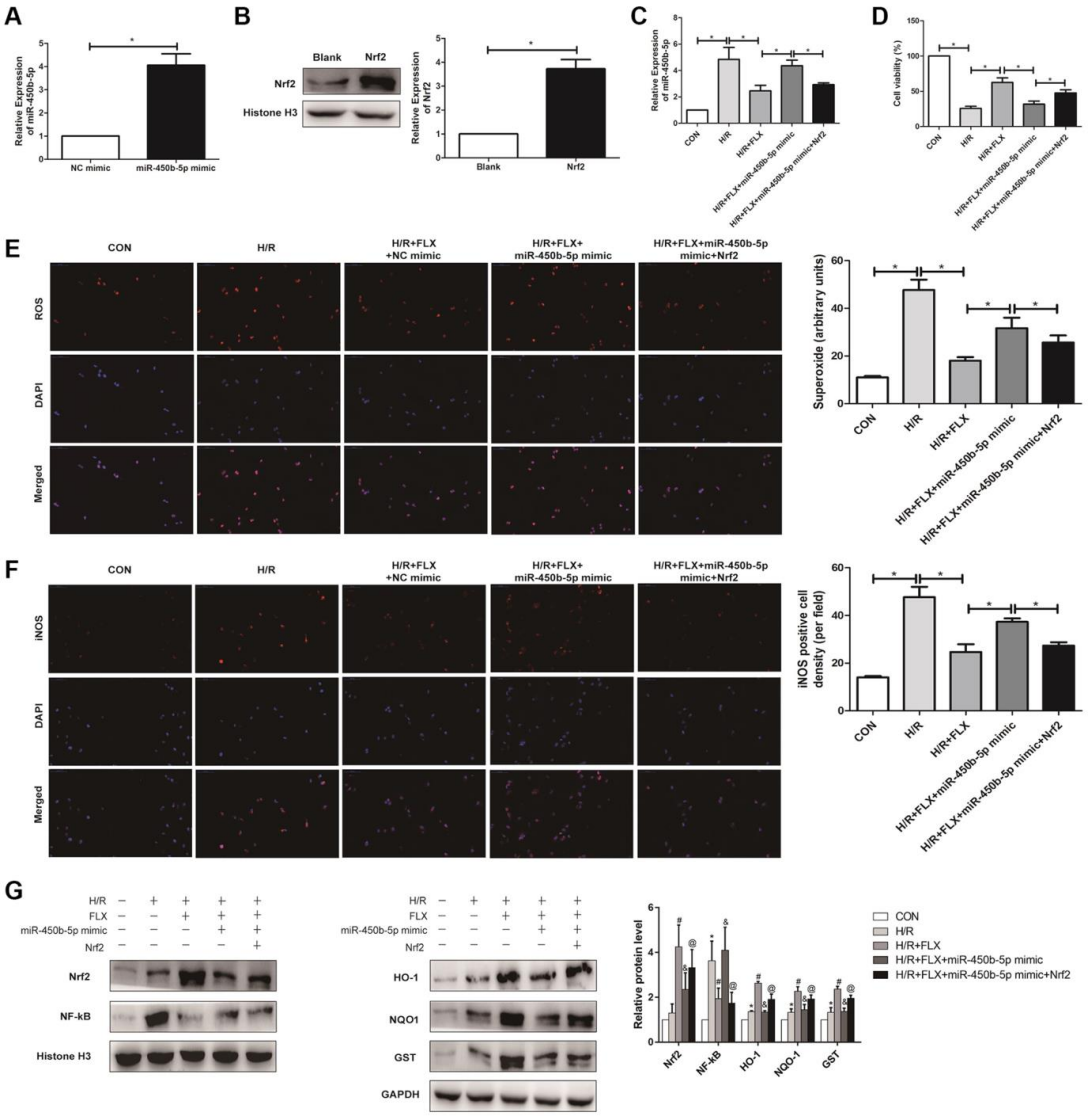
**Fluoxetine up-regulates miR-450b-5p via Nrf2 signaling pathway in HK-2 Hypoxia/Reoxygenation (H/R) model.** (**A**) mRNA expression level of miR-450b-5p after the transfection of miR-450b-5p over-expression vector in HK-2 cell line. (**B**) Protein expression level of Nrf2 after the transfection of miR-450b-5p over-expression vector in HK-2 cell line. (**C**) qRT-PCR analysis showed the expression level of miR-450b-5p from renal tissues in H/R group and H/R+FLX group after the injection of NC mimic, miR-450b-5p mimic or miR-450b-5p mimic+Recombinant protein Nrf2. (**D**) Cell viability of HK-2 in H/R group and H/R+FLX group after the injection of NC mimic, miR-450b-5p mimic or miR-450b-5p mimic+Recombinant protein Nrf2. (**E**) DHE staining of HK-2 cells in H/R group and H/R+FLX group after the injection of NC mimic, miR-450b-5p mimic or miR-450b-5p mimic+Recombinant protein Nrf2. ROS exhibit red fluorescence under fluorescent microscope (magnification ×400). (**F**) Immunohistochemical analysis showed the expression level of iNOS in H/R group and H/R+FLX group after the injection of NC mimic, miR-450b-5p mimic or miR-450b-5p mimic+Recombinant protein Nrf2 in HK-2 cell model (magnification ×400). (**G**) Western Blotting analysis showed the expression level of NF-κB, Nrf2 and Nrf2-dependent antioxidant enzymes, including HO-1, NQO1 and GST in H/R group and H/R+FLX group after the injection of NC mimic, miR-450b-5p mimic or miR-450b-5p mimic+Recombinant protein Nrf2 in HK-2 cell model. Data are expressed as mean ± SD. ^*^significant difference vs. CON group (*P* < 0.05); ^#^significant difference vs. H/R group (*P* < 0.05); ^&^significant difference vs. H/R+FLX group (*P* < 0.05); ^@^significant difference vs. H/R+FLX+miR-450b-5p mimic group (*P* < 0.05).

To further analysis, miR-450b-5p mimic was established in HK-2 cells, and qRT-PCR assay verified the transfection efficiency (Figure 7A). In addition, Western Blot assay showed that the expression level of Nrf2 significantly increased after the administration with 10 nmol recombinant protein Nrf2 in human HK-2 cells (Figure 7B). As shown in Figure 7C, qRT-PCR assay showed that recombinant protein Nrf2 could decrease the expression level of miR-450b-5p, to reverse the oxidative stress induced by HK-2 cells H/R model, in H/R+FLX group. In addition, the results found that miR-450b-5p mimic significantly decreased the cell viability; However, miR-450b-5p mimic+Recombinant Nrf2 dramatically increase the cell viability, compared with miR-450b-5p mimic (Figure 7D). As shown in Figure 7E, immunofluorescence assays showed that the intracellular ROS in FLX pretreatment group was significantly lower than H/R group. In contrast, miR-450b-5p mimic+Recombinant Nrf2 could significantly decrease the excessive generation of ROS compared to miR-450b-5p mimic. Immunohistochemical analysis of iNOS also reflected this trend (Figure 7F).

As shown in Figure 7G, the expression level of Nrf2 in HK-2 cells was significantly increased after administrated with recombinant protein Nrf2. miR-450b-5p mimic reversed the H/R induced-promotion on the expression level of NF-κB. Moreover, miR-450b-5p mimic also reversed H/R induced-inhibition on the expression levels of Nrf2 and its downstream target genes, while recombinant protein Nrf2 further considerably increased the effect of miR-450b-5p mimic on Nrf2, HO-1, NQO1 and GST.

## DISCUSSION

As everyone knows, renal IRI plays a crucial role in the occurrence and development of AKI, which manifests as the deterioration of renal function in a short period [16]. Now, the molecular mechanisms of renal IRI is multifactorial, including ROS, endothelial dysfunction and the apoptotic pathway [17]. However, no effective clinical strategies or therapeutic agents were found for the prevention and treatment of renal ischemic injury. More and more studies reported that the increased production of oxygen-free radicals was considered to be vital factors for renal IRI-induced AKI [18, 19]. Naturally, it was a viable option to reinforcing the cellular antioxidant defense system for the treatment of renal IRI [20].

Numerous studies have suggested that oxidative stress could be regulated by multiple molecular mechanisms [18–20]. Oxygen supply to renal IRI leads to the formation of ROS and inflammatory response, and the overproduction of ROS is considered possible causes of renal IRI [21, 22]. Inducible nitric oxide synthase (iNOS), as one of the main markers of free radical damage, is an important enzyme in the synthesis of NO [23]. Thus, reducing the level of iNOS can reduce the damage of IRI. In the process of lipid peroxidation, MDA, as a biomarker of membrane lipid peroxidation damage, can lead to degeneration of proteins and phospholipids, causing cell contraction and forming cross links [24]. Additionally, previous studies have also found that many antioxidant enzymes, including T-AOC, 8-OHdG and GSH, which conferred the resistance to oxidative stress in various disorders [25, 26]. In our study, we found that a series of oxidative stress biomarkers, such as ROS, iNOS, MDA, 8-OHdG, significantly increased in renal IRI; Nevertheless, FLX could alleviate oxidative stress in renal IRI by the reduction of ROS. Therefore, oxidative stress might be a critical factor in ischemia-induced renal injury, and FLX had a protective effect on renal IRI by antagonizing oxidative stress.

Many scholars have made great efforts to alleviate renal IRI. FLX, considered as one of new antioxidants, has been proven to directly neutralized ROS and down-regulated the activity of NADPH oxidase, thereby reducing the concentration of ROS in the protection of renal IRI [27]. However, no study has as yet worked on the protective role and its mechanism of FLX in renal IRI. Recent study showed that FLX could improve bladder function by reducing the amount of residual urine in the bladder in a mouse model of moderate spinal cord injury [28]. In addition, FLX has been shown to reverse oxidative damage by enhancing antioxidant defense *in vivo* and improving the antioxidant status of cells, thus improving antioxidant capacity after a stress induced decline [29]. Previous studies also showed that FLX could prevent oxidative changes induced by melanoma in mice spleen by its antioxidant system [30, 31]. In the study, we established the experimental model in adolescent male rats, and found that FLX was capable of reducing the levels of Scr and BUN and ameliorating renal impairment related to IRI via using histological and biochemical methods. Hence, the aim of this study was to explore the biological function of FLX in ameliorating male rats renal IRI and HK-2 cells H/R injury and investigate its potential molecular mechanism.

miRNAs have been shown to be one of the potential biomarkers for the diagnosis and treatment of human diseases [32, 33]. One study by Yang et al. has found that miR-144-3p has the potential of protecting myocardial function from IRI through inhibition of TMEM16A Ca^2+^-activated chloride channel [34]. Another study by Liu et al. has suggested that miR-29a in by extracellular vesicles protects brain IRI via NF-κB/NLRP3 axis [35]. Thus, miRNA microarray was used to screen out a total of expressed miRNAs, and found that miR-450b-5p was significantly up-regulated in the renal tissues induced by IRI. MiR-450b-5p has also been confirmed to play important roles in a great deal of inflammatory disease [36]. However, no study about the effects of miR-450b-5p on FLX-induced protective effect for renal IRI was reported. The results found that miR-450b-5p agomir could significantly aggravate the levels of oxidative stress, while tissue antioxidant enzymes were significantly decreased, compared to NC agomir, whether in IRI group or IRI+FLX group. In addition, the results by GEO database and double-luciferase reporter assay showed that miR-450b-5p could directly target Nrf2. Thus, miR-450b-5p might adjust Nrf2 signaling pathway to resist the protective effect FLX in IRI induced renal injury.

Nrf2 is a nuclear transcription factor that regulates oxidative stress damage in a variety of cells, including kidney cells [8, 37]. Moreover, Nrf2 can also directly inhibit the inflammatory response mediated by NF-κB and the release of inflammatory cytokines [38]. Therefore, Nrf2, identified as a potential therapeutic target, could regulate oxidative stress in various disorders [38–40]. In other words, the increase of ROS concentration could inhibit nuclear translocation of Nrf2 and promote the activation of NF-κB. In the present study, FLX could increase the expression level of Nrf2 pathway in the IRI-treated renal injury. Nevertheless, the inhibition of NF-κB activation was probably due to the anti-inflammatory effects of FLX in renal IRI injury. Furthermore, FLX could enhance the activation of Nrf2 and inhibit the expression level of NF-κB in renal injury through the reaction between free radical and anti-inflammatory. Thus, the above results demonstrated that FLX could down-regulated miR-450b-5p to activate Nrf2 signaling pathway to relieve renal IRI injury in rats. However, the underlying mechanisms might be more complex than described here, and other potential mechanisms by which FLX might influence renal IRI might still exist. Further studies on its efficacy and mechanism of action are necessary to provide the protective effect of FLX on renal IRI for its clinical application.

## CONCLUSION

The exogenous supplement of Fluoxetine showed a protective effect on renal IRI, and it promoted its antioxidative effects via down-regulating miR-450b-5p and targeting Nrf2 signaling pathway.
